# Supplementary material for: Cost-effectiveness of antiviral therapy during late pregnancy to prevent perinatal transmission of hepatitis B virus
Source: PeerJ. 2016 Mar 24;4:e1709. doi: 10.7717/peerj.1709 (PMC4811175; doi:10.7717/peerj.1709)
Supplement: Article S1 [file peerj-04-1709-s006.docx]

**Cost-effectiveness of antiviral therapy during late pregnancy to prevent perinatal transmission of hepatitis B virus**

1. **Systematic review and meta-analysis**

*Search*

*Study selection*

*Data extraction*

*Selection*

*Statistical analysis*

1. **Search strategies**

*PubMed*

*CENTRAL*

*EMBASE*

*Chinese Biomedical Literature Database*

*Reference for search strategies*

1. **Systematic review and meta-analysis**

*Search*

We searched PubMed, Embase, Cochrane Central Register of Controlled Trials (CENTRAL), and Chinese Biomedical Literature Database (CBM) for potentially relevant publications without language restriction (updated to September 2014). The detailed search strategy is described in another supplemental file.

*Study selection*

Two authors (WW & WJ) independently screened the records by titles and abstracts. Then they read the full texts of records needing further evaluation. Discrepancies were resolved through group discussion. Controlled studies comparing antiviral therapy plus immunoprophylaxis with immunoprophylaxis only were considered eligible for inclusion. Studies comparing different antiviral therapies along with immunoprophylaxis were also included. The exclusion criteria were as follows: did not test HBsAg of infant at least 6 months after delivery; participants received HBIG during pregnancy; mothers with concomitant liver failure or hepatocellular carcinoma, or co-infected with HIV; reported as abstract only; reviews, case reports, or communications; with inconsistency of data or statistical errors. Duplicate publications were detected by comparing author names, authors’ institutions, locations and settings, numbers of participants, baseline data, and study durations. When more than one publication was identified for the same or overlapping population, we just included the publication with the largest sample size.

*Data extraction*

We extracted the following information from included studies: study design, characteristics of participants, description of interventions, and HBsAg status of infants at least 6 months after delivery. All the information was double-checked.

*Selection*

We identified 21 controlled studies according to our criteria (see Supporting Table). Of these studies, 18 were two-armed studies (5 addressed lamivudine, 12 addressed telbivudine, and 1 addressed tenofovir) and 3 were three-armed studies comparing lamivudine, telbivudine, and control. As the controls were administered with HBIG during pregnancy in one of the three-armed studies, we just extracted data about lamivudine and telbivudine groups in this study. Except one study addressing tenofovir conducted in Tukey, all were conducted in China.

*Statistical analysis*

We performed a network meta-analysis to combine all available direct and indirect comparisons and calculate odds ratios (ORs) for perinatal transmission of HBV. The network meta-analysis uses a multivariate random-effects model. It allows for multiple treatment analysis and I^2^ calculation measuring between-studies heterogeneity. In this analysis, we found no significant between-studies heterogeneity according to I^2^. All analyses were undertaken in STATA with the command *mvmeta*. As only one study with small size addressed tenofovir, we did not include tenofovir in the network meta-analysis.

1. **Search strategies**

*PubMed* (updated to September 2014)：

#1 "Hepatitis B virus"[Mesh] OR "Hepatitis B"[Mesh]

#2 Hepatitis B[Title/Abstract]

#3 (HBV[Title/Abstract]) OR (CHB[Title/Abstract]) OR (Hep B[Title/Abstract])

#4 #1 OR #2 OR #3

#5 "Infectious Disease Transmission, Vertical"[Mesh] OR "Pregnancy"[Mesh]

#6 Pregnan*[Title/Abstract] OR ((mother*[Title/Abstract] OR maternal[Title/Abstract] OR child[Title/Abstract] OR children[Title/Abstract] OR baby[Title/Abstract] OR babies[Title/Abstract] OR infant*[Title/Abstract] OR perinatal[Title/Abstract] OR vertical[Title/Abstract] OR intrauterine[Title/Abstract]) AND (transmission[Title/Abstract] OR infection*[Title/Abstract]))

#7 #5 OR #6

#8 "Lamivudine"[Mesh]

#9 lamivudin*[Title/Abstract] OR epivir[Title/Abstract] OR 3TC[Title/Abstract]

#10 #8 OR #9

#11 "telbivudine" [Supplementary Concept]

#12 telbivudine[Title/Abstract] OR sebivo[Title/Abstract] OR Epavudine[Title/Abstract] OR tyzeka[Title/Abstract] OR telbivudin[Title/Abstract] OR LDT[Title/Abstract]

#13 #11 OR #12

#14 "tenofovir" [Supplementary Concept]

#15 Tenofovir[Title/Abstract] OR TDF[Title/Abstract] OR [Viread](http://www.drugs.com/viread.html)[Title/Abstract]

#16 #14 OR #15

#17 "Antiviral Agents"[Mesh]

#18 (nucleoside analogue*[Title/Abstract]) OR (nucleotide analogue*[Title/Abstract]) OR (antiviral agent*[Title/Abstract]) OR (antiviral drug*[Title/Abstract]) OR (antiviral therap*[Title/Abstract])

#19 #17 OR #18

#20 #10 OR #13 OR #16 OR #19

#21 #4 AND #7 AND #20

*CENTRAL* (updated to September 2014)：

#1 MeSH descriptor: [Hepatitis B virus] explode all trees

#2 MeSH descriptor: [Hepatitis B] explode all trees

#3Hepatitis B:ti,ab,kw (Word variations have been searched)

#4 HBV:ti,ab,kw or CHB:ti,ab,kw or Hep B:ti,ab,kw (Word variations have been searched)

#5 #1 or #2 or #3 or #4

#6 MeSH descriptor: [Infectious Disease Transmission, Vertical] explode all trees

#7 MeSH descriptor: [Pregnancy] explode all trees

#8 Pregnancy:ti,ab,kw (Word variations have been searched)

#9 mother:ti,ab,kw or maternal:ti,ab,kw or child:ti,ab,kw or baby:ti,ab,kw or infant:ti,ab,kw (Word variations have been searched)

#10 perinatal:ti,ab,kw or vertical:ti,ab,kw or intrauterine:ti,ab,kw (Word variations have been searched)

#11 transmission:ti,ab,kw or infection:ti,ab,kw (Word variations have been searched)

#12 #9 or #10

#13 #11 and #12

#14 #6 or #7 or #8 or #13

#15 MeSH descriptor: [Lamivudine] explode all trees

#16 Lamivudine:ti,ab,kw or epivir:ti,ab,kw or 3TC:ti,ab,kw (Word variations have been searched)

#17 Telbivudine:ti,ab,kw or sebivo:ti,ab,kw or Epavudine:ti,ab,kw or tyzeka:ti,ab,kw or LDT:ti,ab,kw (Word variations have been searched)

#18 tenofovir:ti,ab,kw or TDF:ti,ab,kw or Viread:ti,ab,kw (Word variations have been searched)

#19 MeSH descriptor: [Antiviral Agents] explode all trees

#20 nucleoside analogue:ti,ab,kw or antiviral agent:ti,ab,kw or antiviral drug:ti,ab,kw (Word variations have been searched)

#21 antiviral therapy:ti,ab,kw (Word variations have been searched)

#22 #15 or #16 or #17 or #18 or #19 or #20

#23 #5 and #14 and #22

*EMBASE* (updated to September 2014):

#55 #7 AND #26 AND #54

#54 #27 OR #28 OR #29 OR #30 OR #31 OR #32 OR #33 OR #34 OR #35 OR #36 OR #37 OR #38 OR #39 OR #40 OR #41 OR #42 OR #43 OR #44 OR #45 OR #46 OR #47 OR #53

#53 #48 AND #52

#52 #49 OR #50 OR #51

#51 therap*:ab,ti AND [embase]/lim

#50 drug*:ab,ti AND [embase]/lim

#49 agent*:ab,ti AND [embase]/lim

#48 antiviral:ab,ti AND [embase]/lim

#47 'nucleotide analogues':ab,ti AND [embase]/lim

#46 'nucleotide analogue':ab,ti AND [embase]/lim

#45 'nucleoside analogues':ab,ti AND [embase]/lim

#44 'nucleoside analogue':ab,ti AND [embase]/lim

#43 'nucleoside analog'/exp AND [embase]/lim

#42 'antivirus agent'/exp AND [embase]/lim

#41 viread:ab,ti AND [embase]/lim

#40 tdf:ab,ti AND [embase]/lim

#39 tenofovir:ab,ti AND [embase]/lim

#38 'tenofovir'/exp AND [embase]/lim

#37 ldt:ab,ti AND [embase]/lim

#36 telbivudin:ab,ti AND [embase]/lim

#35 tyzeka:ab,ti AND [embase]/lim

#34 epavudine:ab,ti AND [embase]/lim

#33 sebivo:ab,ti AND [embase]/lim

#32 telbivudine:ab,ti AND [embase]/lim

#31 'telbivudine'/exp AND [embase]/lim

#30 3tc:ab,ti AND [embase]/lim

#29 epivir:ab,ti AND [embase]/lim

#28 lamivudin*:ab,ti AND [embase]/lim

#27 'lamivudine'/exp AND [embase]/lim

#26 #8 OR #9 OR #10 OR #25

#25 #21 AND #24

#24 #22 OR #23

#23 infection*:ab,ti AND [embase]/lim

#22 transmission:ab,ti AND [embase]/lim

#21 #11 OR #12 OR #13 OR #14 OR #15 OR #16 OR #17 OR #18 OR #19 OR #20

#20 intrauterine:ab,ti AND [embase]/lim

#19 vertical:ab,ti AND [embase]/lim

#18 perinatal:ab,ti AND [embase]/lim

#17 infant*:ab,ti AND [embase]/lim

#16 babies:ab,ti AND [embase]/lim

#15 baby:ab,ti AND [embase]/lim

#14 children:ab,ti AND [embase]/lim

#13 child:ab,ti AND [embase]/lim

#12 maternal:ab,ti AND [embase]/lim

#11 mother*:ab,ti AND [embase]/lim

#10 pregnan*:ab,ti AND [embase]/lim

#9 'pregnancy'/exp AND [embase]/lim

#8 'vertical transmission'/exp AND [embase]/lim

#7 #1 OR #2 OR #3 OR #4 OR #5 OR #6

#6 'hepatitis b':ab,ti AND [embase]/lim

#5 'hep b':ab,ti AND [embase]/lim

#4 chb:ab,ti AND [embase]/lim

#3 hbv:ab,ti AND [embase]/lim

#2 'hepatitis b'/exp AND [embase]/lim

#1 'hepatitis b virus'/exp AND [embase]/lim

*Chinese Biomedical Literature Database* (updated to September 2014)：

#33 #32 and #19 and #7
#32 #31 or #30 or #29 or #28 or #27 or #26 or #25 or #24 or #23 or #22 or #21 or #20
#31 缺省:韦瑞德
#30 缺省:素比伏
#29 缺省:贺普丁
#28 缺省:tenofovir
#27 缺省:telbivudine
#26 缺省:Lamivudine
#25 缺省:泰诺福韦
#24 缺省:替诺福韦
#23 缺省:替比夫定
#22 缺省:拉米夫定
#21 主题词:抗病毒药/全部树/全部副主题词
#20 主题词:拉米夫定/全部树/全部副主题词
#19 #18 or #17 or #16 or #15 or #14 or #13 or #12 or #11 or #10 or #9 or #8
#18 缺省:婴儿
#17 缺省:胎儿
#16 缺省:新生儿
#15 缺省:宫内
#14 缺省:孕妇
#13 缺省:孕期
#12 缺省:围产期
#11 缺省:垂直传播
#10 缺省:母婴
#9 主题词:孕妇/全部树/全部副主题词
#8 主题词:疾病传播, 垂直/全部树/全部副主题词
#7 #6 or #5 or #4 or #3 or #2 or #1
#6 缺省:HBV
#5 缺省:乙肝
#4 缺省:乙型病毒性
#3 缺省:乙型肝炎
#2 主题词:肝炎病毒, 乙型/全部树/全部副主题词
#1 主题词:肝炎, 乙型, 慢性/全部树/全部副主题词

*Reference for search strategies:*

1. Eke Ahizechukwu C, Eke Uzoamaka A, Uchenna E. Hepatitis B immunoglobulin during pregnancy for the prevention of mother to child transmission of hepatitis B virus. Cochrane Database of Systematic Reviews. John Wiley & Sons, Ltd, 2010.
2. Lee C, Gong Y, Brok J, et al. Hepatitis B immunisation for newborn infants of hepatitis B surface antigen-positive mothers[J]. Cochrane Database Syst Rev, 2006, (2): CD004790.
3. Mumtaz K, Ahmed Umair S, Zuberi Nadeem F, et al. Lamivudine during pregnancy for preventing hepatitis B virus infection in newborns. Cochrane Database of Systematic Reviews. John Wiley & Sons, Ltd, 2010.
4. Spaulding A, Rutherford GW, Siegfried N. Tenofovir or zidovudine in three-drug combination therapy with one nucleoside reverse transcriptase inhibitor and one non-nucleoside reverse transcriptase inhibitor for initial treatment of HIV infection in antiretroviral-naive individuals[J]. Cochrane Database Syst Rev, 2010, (10): CD008740.
5. Zhao S, Tang L, Fan X, et al. Telbivudine for chronic hepatitis B. Cochrane Database of Systematic Reviews. John Wiley & Sons, Ltd, 2010.
