# Supplementary material for: Cost-effectiveness of antiviral therapy during late pregnancy to prevent perinatal transmission of hepatitis B virus
Source: PeerJ. 2016 Mar 24;4:e1709. doi: 10.7717/peerj.1709 (PMC4811175; doi:10.7717/peerj.1709)
Supplement: Table S1 [file peerj-04-1709-s007.docx]

Controlled studies evaluating antiviral therapy plus immunoprophylaxis for protection of perinatal transmission of hepatitis B virus.

| Ref. | Study | Design | Mothers (T/C) | HBeAg (T/C) | HBV-DNA (copies/ml) | Start time | End time | Vaccine | HBIG | HBsAg at 6-7 mo (N_T_, n_T_\|N_C_, n_C_) | HBsAg at 12 mo (N_T_, n_T_\|N_C_, n_C_) |
| --- | --- | --- | --- | --- | --- | --- | --- | --- | --- | --- | --- |
| LAM |  |  |  |  |  |  |  |  |  |  |  |
| 26 | Feng, 2007 | NRS | 48/42 | 48/42 | >6 log | 28 w | 4 w after delivery | 10 μg, 0-1-6 | ≥100 IU, 1d | 48, 7\|42, 16 | na |
| 27 | Guo, 2008 | RCT | 70/40 | 70/40 | na | 28 w | after delivery | 10 μg, 0-1-6 | 200 IU, 1d, 15d | 70, 3\|40, 13 | na |
| 28 | Xu, 2009 | RCT | 61/61 | 61/61 | >9 log | 30-34 w | 4 w after delivery | na, 0-1-6 | 200 IU, 1d | 56, 3\|59, 6 | 56, 3\|59, 5 |
| 29 | Zhang, 2010 | NRS | 50/50 | 50/50 | >6 log | 28 w | 4 w after delivery | 10 μg, 0-1-6 | 200 IU, 1d, 30d | 50, 3\|50, 16 | 50, 1\|50, 8 |
| 37 | Wang, 2012^#^ | NRS | 32/27 | 32/27 | >7 log | 28 w | 4 w after delivery | 10 μg, 0-1-6 | 100 IU, 1d, 30d | na | 32, 1\|27, 5 |
| 43 | Zeng, 2013 | RCT | 90/30 | 90/30 | >5 log | 28 w | 0, 4, or 6 w after delivery | 10 μg, 0-1-6 | 200 IU, 1d | na | 90, 0\|30, 3 |
| 44 | Yu, 2014^‡^ | NRS | 154/233 | 154/233 | >6 log | 8-32 w | after delivery | 20 μg, 0-1-6 | 200 IU, 1d, 15d | na | 154, 0\|233, 0 |
| 45 | Zhang, 2014^#^ | NRS | 55/374 | 55/374 | >6 log | 28-30 w | 4 w after delivery | 10 μg, 0-1-6 | 200 IU, 1d, 15d | na | 52, 0\|352, 10 |
| LdT |  |  |  |  |  |  |  |  |  |  |  |
| 30 | Zhao, 2010 | RCT | 30/30 | 30/30 | na | 28 w | after delivery | 10 μg, 0-1-6 | 200 IU, 1d, 30d | na | 30, 1\|30, 3 |
| 31 | Guo, 2011 | RCT | 25/25 | 25/25 | >7 log | 28 w | 4 w after delivery | 20 μg, 0-1-6 | 200 IU, 1d, 30d | 28, 6\|26, 6 | 28, 4\|26, 11 |
| 32 | Han, 2011 | NRS | 135/94 | 135/94 | >7 log | 20-32 w | 4 w after delivery | 20 μg, 0-1-6 | 200 IU, 1d, 15d | 132, 0\|88, 7 | na |
| 33 | Yao, 2011 | NRS | 28/30 | na^§^ | >6 log | 28 w | 4 w after delivery | 10 μg, 0-1-6 | 200 IU, 1d, 30d | 28, 1\|30, 5 | 28, 0\|30, 4 |
| 34 | Zhou, 2011 | NRS | 36/40 | 34/36 | >7 log | early pregnancy | > 1 year after delivery | 20 μg, 0-1-6 | na | na | 33, 1\|34, 6 |
| 35 | Pan, 2012 | NRS | 53/35 | 53/35 | >6 log | 12-30 w | continue | 20 μg, 0-1-6 | 200 IU, 1d, 15d | 52, 0\|32, 3 | na |
| 36 | Peng, 2012 | NRS | 40/40 | 40/40 | >6 log | 28 w | after delivery | 10 μg, 0-1-6 | 200 IU, 1d, 30d | 40, 1\|40, 10 | 40, 1\|40, 10 |
| 37 | Wang, 2012^#^ | NRS | 28/27 | 28/27 | >7 log | 28 w | 4 w after delivery | 10 μg, 0-1-6 | 100 IU, 1d, 30d | na | 28, 0\|27, 5 |
| 38 | Wang, 2012 | NRS | 47/198 | 47/198 | >5 log | 12-28 w | after delivery | 10 μg, 0-1-6 | 200 IU, 1d | 47, 0\|198, 20 | na |
| 39 | Bai, 2013 | NRS | 30/30 | 30/30 | >7 log | 28-32 w | 4 w after delivery | 10 μg, 0-1-6 | 200 IU, 1d, 30d | 30, 0\|30, 4 | na |
| 40 | Jiang, 2013 | NRS | 65/51 | 65/51 | >5 log | 26-30 w | continue | 20 μg, 0-1-6 | 200 IU, na | 65, 1\|51, 8 | na |
| 41 | Sun, 2013 | NRS | 38/42 | 38/42 | >7 log | 12 w | 12 w after delivery | 20 μg, 0-1-6 | 200 IU, 1d, 30d | 38, 0\|42, 6 | na |
| 42 | Wu, 2013 | NRS | 69/15 | 69/15 | >6 log^*^ | 24-33 w | na | na, 0-1-6 | na | 70, 0\|15, 1 | 70, 0\|15, 1 |
| 45 | Zhang, 2014^#^ | NRS | 263/374 | 263/374 | >6 log | 28-30 w | 4 w after delivery | 10 μg, 0-1-6 | 200 IU, 1d, 15d | na | 257, 0\|352, 10 |
| TDF |  |  |  |  |  |  |  |  |  |  |  |
| 16 | Celen, 2013 | NRS | 21/24 | 21/24 | >7 log | 18-27 w | 4 w after delivery | 20 μg, 1-2-6 | 200 IU, 1d | 21, 0\|23, 2 | na |

T: antiviral treatment; C: control; N: number of infants followed for at least 6 months; n: number of infants positive for HBsAg; HBsAg: hepatitis B surface antigen; HBeAg: hepatitis B e antigen; HBV: hepatitis B virus; HBIG: hepatitis B immunoglobulin; RCT: randomised controlled trial; NRS: non-randomised studies; LAM: lamivudine; LdT: telbivudine; TDF: tenofovir; na: not available.

^#^Three-armed studies comparing lamivudine, telbivudine, and control.

^‡^Control group was treated with telbivudine during pregnancy.

^§^42 positive for HBeAg and 16 negative for HBeAg overall.

^*^Presented as IU/ml.
